# Silver nanoparticles administered to chicken affect *VEGFA* and *FGF2* gene expression in breast muscle and heart

**DOI:** 10.1186/1556-276X-7-418

**Published:** 2012-07-24

**Authors:** Anna Hotowy, Ewa Sawosz, Lane Pineda, Filip Sawosz, Marta Grodzik, André Chwalibog

**Affiliations:** 1 Department of Basic Animal and Veterinary Sciences, University of Copenhagen, Groennegaardsvej 3, Frederiksberg, 1870, Denmark; 2 Nanobiotechnology Laboratory, Warsaw University of Life Sciences, Warsaw, 02-786, Poland

**Keywords:** Silver nanoparticles, *VEGFA*, *FGF2*, Muscle, Heart, Chicken

## Abstract

Nanoparticles of colloidal silver (AgNano) can influence gene expression. Concerning trials of AgNano application in poultry nutrition, it is useful to reveal whether they affect the expression of genes crucial for bird development. AgNano were administered to broiler chickens as a water solution in two concentrations (10 and 20 ppm). After dissection of the birds, breast muscles and hearts were collected. Gene expression of *FGF2* and *VEGFA* on the mRNA and protein levels were evaluated using quantitative polymerase chain reaction and enzyme-linked immunosorbent assay methods. The results for gene expression in the breast muscle revealed changes on the mRNA level (*FGF2* was up-regulated, *P* < 0.05) but not on the protein level. In the heart, 20 ppm of silver nanoparticles in drinking water increased the expression of *VEGFA* (*P* < 0.05), at the same time decreasing *FGF2* expression both on the transcriptional and translational levels. Changes in the expression of these genes may lead to histological changes, but this needs to be proven using histological and immunohistochemical examination of tissues. In general, we showed that AgNano application in poultry feeding influences the expression of *FGF2* and *VEGFA* genes on the mRNA and protein levels in growing chicken.

## Background

Nanoparticles of colloidal silver (AgNano) are small enough to penetrate into the cell and subsequently into the nucleus. Direct interaction between them and DNA molecules or DNA-related proteins leads to alterations in gene expression profiles [1]. Clear evidence exists that AgNano can modify gene expression in animal models both *in vivo* and *in vitro*[2-5]. Taking into account the first trials of silver nanoparticle application in poultry production as an antimicrobial and nutritional agent [6], it is necessary to evaluate their effects on gene expression. From a nutritional point of view, it would be interesting to investigate genes with a crucial meaning for the muscle development in birds. We have focused on two genes, namely basic fibroblast growth factor 2 (*FGF2*) and vascular endothelial growth factor A (*VEGFA*).

FGF2 consists of low and high molecular weight protein isoforms, localised to different cellular compartments, indicating unique biological activity *in vivo* and *in vitro*[7,8]. It has mitogenic activity on a variety of mesodermal cells, including myoblasts [9]. FGF2 is implicated in early vertebrate embryogenesis [10] and tissue regeneration [11]. The immunolocalisation of FGF2 demonstrates that the spatial pattern of FGF2 localisation is highly specific. In the developing chick embryo, FGF2 has been localised in the myocardium, the somite myotome and limb bud muscles [12]. Experiments in pigs also suggest that *FGF2* might be involved in skeletal muscle development during foetal and early postnatal life [13]. In the heart, FGF2 isoforms have distinct roles in many pathological conditions, including cardiac hypertrophy, ischemia-reperfusion injury and atherosclerosis [7].

VEGFA is essential for early development of the vasculature to the extent that inactivation of even a single allele of the *VEGFA* gene results in embryonic lethality [14,15]; furthermore, VEGFA is essential in regulating postnatal muscle capillarity. Cardiac and skeletal muscles of adult *VEGFA*-deficient animals exhibit a major intolerance to aerobic exercise [16].

Moreover, VEGFA positively influences dystrophic skeletal muscle repair, improving their vascularity and endogenous regeneration while lowering fibrosis; however, its uncontrolled expression causes unfavourable changes in muscle morphology [17,18]. VEGFA exerts action not only locally. It can mobilise endothelial progenitor cells from the bone marrow even to distant sites of neovascularisation [19].

Nutrition, in the case of poultry, can also comprise *in ovo* feeding that can stimulate intestinal development by enhancing villi development, thus increasing the intestinal capacity to digest and absorb nutrients, which provides a basis for muscle growth [20]. Moreover, both VEGFA and FGF2 injected into the vitelline vein of the chicken embryo stimulate myocardial vascularization, each of the proteins in a different way [21].

Few studies can be found on FGF2 and VEGFA expression, and their action in chicken embryos [12,21], but there is no information whether AgNano can affect expression of these genes in embryonic and growing chicken as well. The purpose of this study was to assess the influence of AgNano delivered *in ovo* and *in vivo* on the expression of the abovementioned genes in chicken and to indicate further research directions, which could reveal possible AgNano applications.

## Methods

Experiments, conducted during the prenatal and postnatal growth periods of broiler chickens, were carried out in accordance with the Animal Experimentation Act in Denmark (Law No. 726, September 1993).

### Nanoparticles

The hydrocolloid of AgNano (Nano-Tech, Warsaw, Poland) was produced by a patented non-explosive high-voltage method from high-purity metals (99.9999%) and high-purity demineralised water. The colloid contained 50 ppm of Ag nanoparticles, with a particle size ranging from 2 to 6 nm, based on transmission electron microscope (TEM) evaluation as described by Chwalibog et al. [22]. Experimental drinking water solutions used in the experiments were prepared by diluting the original concentration of the AgNano solution (50 ppm) in distilled water. The concentrations were selected based on effective dose recorded in weanling pigs (20 ppm of AgNano) [23] and in broilers (10 ppm; LP, unpublished data), and on economically relevant level.

### *In ovo* treatments

Fertile eggs from Ross × Ross 308 breeder hens were obtained from a commercial hatchery and transported to the experimental farm of the University of Copenhagen. Upon arrival, the eggs were numbered, weighed and consequently treated according to the following descriptions: (1) non-injected control (*n* = 240), (2) AgNano injected (10 ppm of colloidal AgNano, *n* = 96) and (3) AgNano injected (20 ppm of colloidal AgNano, *n* = 96). The eggs were injected with 0.3 ml of the AgNano solutions (10 or 20 ppm) into the air sac at the blunt end of the albumen using sterile 1-ml tuberculin syringes with a 27-gauge needle. Before and after the injection, the shell was disinfected with ethanol. Afterwards, the hole was sealed with hypoallergenic tape and the eggs were placed in an incubator. The eggs were incubated for 21 days under standard conditions (temperature 37.8°C, 55% humidity, turned once per hour during the first 18 days, and at a temperature of 37°C and 60% humidity from day 19 until hatching).

### *In vivo* treatments

**Figure 1:**
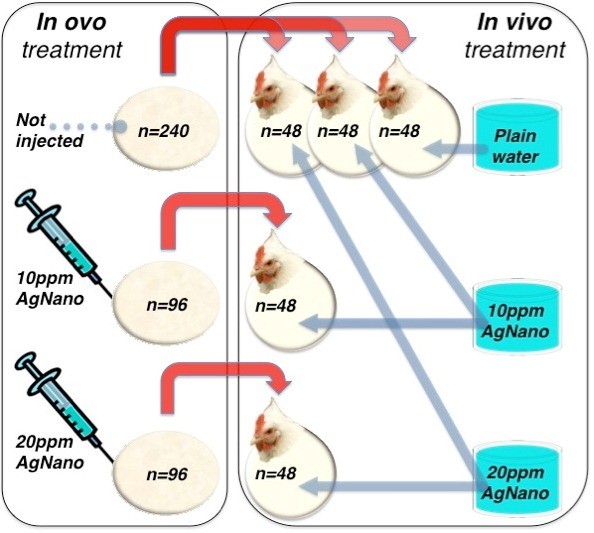
**Experimental design.** Red arrows indicate random selection of the birds after hatching, from the *in ovo* part (eggs not injected and injected with AgNano) to the *in vivo* part (broilers treated with AgNano drinking solution) of the experiment.

For the post-hatching experiment, five groups of 48 chickens were formed. Three groups were created from the non-injected control group, and two additional groups were formed from the groups injected with 10 and 20 ppm of AgNano (Figure 1). Chicks for each group were randomly selected.

After hatching, for the first 6 days, the chicks were kept in pens furnished with a heating lamp. The temperature inside the brooding pen was 30°C to 33°C, and the lighting program was 23L:1D (L = light, D = darkness).

The 6-day transition period was applied to assure that the chicks get used to the environmental conditions before the actual experiment starts and to exclude a change of these conditions as a potential, additional factor influencing the results.

On day 7, 24 chicks from each group were randomly selected, weighed, leg banded and transferred to metabolic cages (0.5 m × 0.5 m × 0.5 m) with four birds per cage allocated into six replications. The room temperature from 7 to 35 days was 22°C with the following lighting program: days 7 to 13, 20L:4D; days 14 to 20, 21L:3D; days 21 to 27, 22L:2D and days 28 to 35, 23L:1D.

Throughout the experiment, the birds were fed *ad libitum* with a commercial diet. For the first 6 days after hatching, they obtained a commercial mixture containing 18.5% crude protein, 4.4% crude fat, 5.0% crude fibre, 5.8% ash, 10.3 g/kg lysine, 4.1 g/kg methionine and 3.4 g/kg cysteine. In the following 4 weeks, they were given another mixture containing 17.6% crude protein, 3.3% crude fat, 3.8% crude fibre, 5.2% ash, 8.7 g/kg lysine, 4.1 g/kg methionine and 3.2 g/kg cysteine. Both mixtures were produced by DLG (Axelborg, Copenhagen, Denmark). The birds had free access to water containing 0, 10 or 20 ppm of colloidal AgNano offered for 4 weeks after an initial 6-day-long transition period during the brooding period. The broilers' body weight, feed consumption and water intake were recorded at the beginning and end of each week starting at day 7 until 35 days of age.

### Dissection procedure

After 4 weeks, the broilers were weighed and euthanized and the samples were collected. The hearts were weighed, and relative heart weight was calculated as the heart weight divided by the body weight, expressed as percentage.

Tissues were put into labelled cryo-tubes and immediately frozen in liquid nitrogen. Afterwards, all the tubes with frozen organs belonging to the same broiler were placed in one plastic bag labelled with the ID number of the bird and stored in the deep freezer (−80°C) until further analysis.

### Gene expression on the mRNA level

Gene expression on the mRNA level was measured in breast muscle and heart samples using quantitative polymerase chain reaction method. The tissues (breast muscle and heart) were homogenised in TRIzol reagent (Invitrogen, Carlsbad, CA, USA) using TissueLyser II (Qiagen, Venlo, Netherlands), and total RNA was extracted according to the manufacturer's instructions. The RNA samples were purified using the SV Total RNA Isolation System (Promega, Fitchburg, WI, USA). The total RNA concentration was measured using a NanoDrop ND 1000 Spectrophotometer (NanoDrop Technologies, Wilmington, DE, USA). Two micrograms of total RNA were reverse transcribed using reverse transcriptase (Promega), oligo-dT and random primers (TAG Copenhagen A/S Symbion, Copenhagen, Denmark), after which real-time PCR was performed with cDNAs and gene-specific primer pairs (TAG Copenhagen A/S Symbion; Table 1) mixed with LightCycler®480 SYBR Green I Master (Roche Diagnostics, Basel, Switzerland) using a LightCycler®480 system (Roche Diagnostics). The samples were first denatured for 5 min at 95°C and then amplified using 45 cycles of 10 s at 95°C (denaturation), 10 s at 60°C to 62°C (annealing) and 9 s at 72°C (elongation) followed by quantitation. The reaction was performed in triplicate for each cDNA. For all analyses, relative quantification was applied, and actin beta (*ACTB*) and elongation factor 1 alpha 2 (*EEF1A2*) were used as the housekeeping genes*.*

**Table 1:** Primer sequences for the investigated genes

**Gene symbol**	**Gene ID**^**a**^	**Size (bp)**	**Primer sequence (5’ to 3’)**
*ACTB*	396526	*ca.* 169	forward: GTCCACCTTCCAGCAGATGT
			reverse: ATAAAGCCATGCCAATCTCG
*EEF1A2*	419244	*ca.* 85	forward: AGCAGACTTTGTGACCTTGCC
			reverse: TGACATGAGACAGACGGTTGC
*FGF2*	396413	*ca.* 151	forward: GGCACTGAAATGTGCAACAG
			reverse: TCCAGGTCCAGTTTTTGGTC
*VEGFA*	395909	*ca.* 194	forward: TGAGGGCCTAGAATGTGTCC
			reverse: TCTTTTGACCCTTCCCCTTT

^a^NCBI resources.

### Gene expression on the protein level

To estimate protein expression, enzyme-linked immunosorbent assay (ELISA) was applied*.* Samples from the control group receiving water and groups receiving 20 ppm of AgNano were chosen. Samples of heart and muscle tissues were homogenised in chilled RIPA buffer (Sigma-Aldrich, St. Louis, MO, USA) mixed with protease and phosphatase inhibitors (Protease Inhibitor Cocktail for use with mammalian cell and tissue extracts, DMSO solution, Sigma-Aldrich, and Phosphatase Inhibitor Cocktail 2, Sigma-Aldrich), using TissueLyser II (Qiagen). Then, samples were kept at 4°C for 1 h while slowly mixing. Afterwards, the samples were centrifuged at 4°C (30 min, 14,000 rpm). The supernatant was aliquoted into Eppendorf tubes to avoid frequent freeze/thaw cycles and frozen at −20°C. Each aliquot was used for determining the protein concentration (Total Protein Kit, MicroLowry, Peterson's Modification, Sigma-Aldrich) and for ELISA tests on the same day.

Kits for gallinaceous Fibroblast Growth Factor 2 and Vascular Endothelial Cell Growth Factor A (USCN Life Science, Wuhan, China) were used. Reagents and plates were prepared according to the manufacturer's instructions. The level of absorption was measured using an Infinite M200 microplate reader (TECAN, Crailsheim, Germany) at a wavelength of 450 nm. All samples were measured in duplicate.

### Statistical analysis

Analysis of the data was carried out using *t*-tests and one-way analysis of variance (ANOVA) tests - generalized linear model procedure in SAS 9.2 (SAS Windows, 2002–2008, version 9.2, SAS Institute Inc., Cary, NC, USA). Values differing at *P* < 0.05 were considered significant.

## Results

There were no significant differences in body weight, heart weight and relative heart weight between experimental groups (Table 2).

**Table 2:** Body and heart weight of chickens treated with different levels of AgNano

**Weight**	**Treatment**					**ANOVA**	
	**Control**	**10 ppm AgNano**	**20 ppm AgNano**	**10 ppm AgNano**^**b**^	**20 ppm AgNano**^**b**^	**SEM**	***P*value**
Body (kg)	1.578	1.684	1.725	1.436	1.545	0.0455	0.2853
Heart (g)	6.52	7.00	6.80	6.14	6.23	0.299	0.8937
Heart^a^ (relative, %)	0.407	0.413	0.392	0.427	0.406	0.0124	0.9427

^a^Values were calculated as the heart weight divided by the body weight, expressed as percentage; ^b^eggs were *in ovo* injected; the level of significance is *P* < 0.05. AgNano, nanoparticles of colloidal silver; ANOVA, analysis of variance; SEM, standard error of the mean.

### Gene expression

#### Breast muscle

The expression of *FGF2*, normalised to *ACTB*, was higher in all treatment groups than in the control group; however, differences between treatments were not significant. Regarding *EEF1A2*, significant differences were observed only between the control and the group which obtained the higher dose (20 ppm) of AgNano in the drinking water but had not been injected *in ovo* (Table 3).

**Table 3:** Relative gene expression in the muscle tissue of chickens treated with different levels of AgNano

**Gene symbol**	**Treatment**					**ANOVA**	
	**Control**	**10 ppm AgNano**	**20 ppm AgNano**	**10 ppm AgNano**^**c**^	**20 ppm AgNano**^**c**^	**SEM**	***P *value**
*FGF2*^a^	0.67^†^	1.28^‡^	1.44^‡^	1.05^‡^	1.23^‡^	0.070	0.0003
*FGF2*^b^	0.60^†^	0.98	1.38^‡^	0.95	0.93	0.068	0.0024
*VEGFA*^a^	1.39	1.08^†^	1.03^†^	1.13	1.60^‡^	0.064	0.0057
*VEGFA*^b^	1.28	1.02^†^	0.95^†^	1.01	1.28^‡^	0.055	0.0072

^a^Normalised to *ACTB*; ^b^normalised to *EEF1A2*; ^c^eggs were *in ovo* injected; ^†,‡^different symbols indicate statistically significant differences (*P* < 0.05). AgNano, nanoparticles of colloidal silver; ANOVA, analysis of variance; SEM, standard error of the mean.

When *VEGFA* was examined, the level of expression in the breast muscle was higher in the group that had been injected *in ovo* and obtained 20 ppm of AgNano in the drinking water, compared to both non-injected groups receiving different solutions of AgNano in the drinking water. At the same time, the expression of this gene did not differ between the two injected groups and the untreated control group receiving water (Table 3).

#### Heart

Regarding *FGF2* expression levels in the heart, when *ACTB* was used as the housekeeping gene, a significant difference was observed between the control and the group injected *in ovo* and treated with 20 ppm of AgNano in the drinking water. The expression of *FGF2* normalised to *EEF1A2* revealed no significant differences between the experimental groups (Table 4).

**Table 4:** Relative gene expression in the heart of chickens treated with different levels of AgNano

**Gene symbol**	**Treatment**					**ANOVA**	
	**Control**	**10 ppm AgNano**	**20 ppm AgNano**	**10 ppm AgNano**^**c**^	**20 ppm AgNano**^**c**^	**SEM**	***P *value**
*FGF2*^a^	0.97^†^	0.85	0.79	0.84	0.69^‡^	0.028	0.0443
*FGF2*^b^	1.47	1.32	1.34	1.15	1.31	0.054	0.5839
*VEGFA*^a^	0.59	0.68	0.78	0.73	0.61	0.026	0.0888
*VEGFA*^b^	0.75^†^	0.93	1.12^‡^	0.84	1.14^‡^	0.044	0.0027

^a^Normalised to *ACTB*; ^b^normalised to *EEF1A2*; ^c^eggs were *in ovo* injected; ^†,‡^different symbols indicate statistically significant differences (*P* < 0.05). AgNano, nanoparticles of colloidal silver; ANOVA, analysis of variance; SEM, standard error of the mean.

In the case of *VEGFA* normalised to *ACTB*, no significant differences could be reported between the experimental groups. The high level of AgNano (20 ppm) given in the drinking water increased the expression of *VEGFA* normalised to *EEF1A2* in both the injected and non-injected treatment groups (Table 4).

### Protein expression

**Figure 2:**
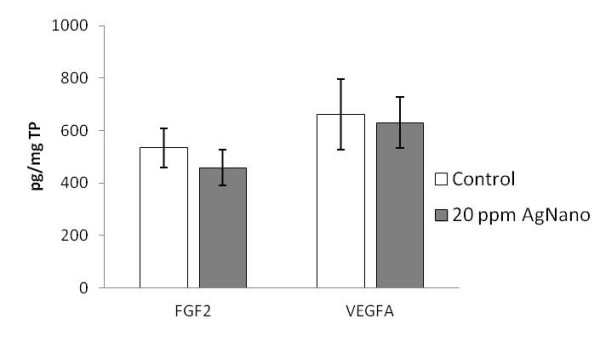
**Protein expression in the breast muscle.** The vertical axis shows the concentration of the target protein in picograms per milligram of total protein (TP). Error bars indicate standard deviation (SD). The level of significance is *P* < 0.05.

Concerning FGF2 and VEGFA on the protein level, no significant differences were found between treatments in the muscle tissue (Figure 2).

**Figure 3:**
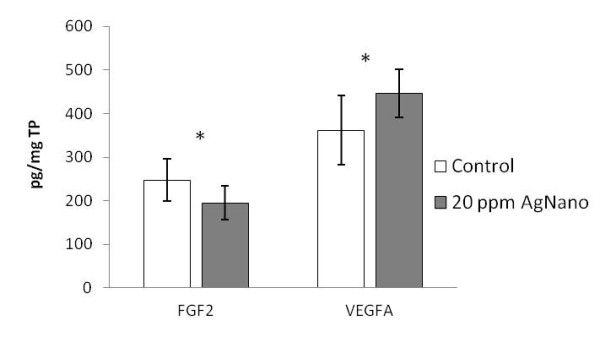
**Protein expression in the heart.** The vertical axis shows the concentration of the target protein in picograms per milligram of total protein (TP). Error bars indicate standard deviation (SD). Asterisks indicate statistically significant differences (*P* < 0.05).

In the heart, the level of FGF2 protein after AgNano administration was lower, whereas the level of VEGFA protein was higher in the group treated with 20 ppm of AgNano, compared to the control group (Figure 3).

## Discussion

Due to the tissue specificity of the reference genes [24-27], the results for each examined target gene are not identical relative to the individual reference genes (especially in the heart). However, for both housekeeping genes, the relative levels of target gene expression presented almost the same pattern of changes.

Our results suggest that, in the case of chicken breast muscle tissue, oral AgNano administration influences *FGF2* and *VEGFA* expression on the mRNA level, without leading to changes on the protein level. It could be suspected, at least in the case of FGF2, that such a difference might be caused by an unspecific isoform of this protein. Currently, only low molecular weight recombinant FGF2 protein is available on the market. However, the FGF2 antibody is not differentially specific against the two isoforms [7]. Assuming this, our results could mean that all the histological changes (like multipotent mesenchymal cell differentiation, satellite cell proliferation and angiogenesis) that have been noted in previous *in vitro* and *in vivo* studies [28-32] should not be expected in our case, or at least may not be caused directly by changes of FGF2 and VEGFA protein expression. This effect could be considered beneficial in terms of angiogenesis, which is a fundamental process affecting tumour growth and metastasis [19,33-37]. Nevertheless, this remains to be clarified by applying histological and immunohistochemical methods of examination of the tissue.

The opposite effect of AgNano administration was observed in the case of the cardiac muscle, where, without any impact on relative heart weight (Table 2), the expression of both genes was affected.

*FGF2* expression on the mRNA level was down-regulated in the *in ovo* injected group which obtained 20 ppm of AgNano in the drinking water, compared to the control group. Similar results were noted regarding protein expression, where the FGF2 level was lower in the AgNano-treated group compared to the control group. The results obtained for FGF2 in our case are not in agreement with the findings of Bougioukas et al. [38], which suggested possible neovascularisation in the heart caused by this protein. However, according to another study, the expression of this gene remains unchanged during angiogenesis caused by bradycardia, suggesting that FGF2 does not play a direct role in this process [14]. On the other hand, some evidence exists that FGF2 (the high molecular weight isoform) can prevent endothelial cell migration and angiogenesis [39] and promote cardiac myocyte hypertrophy [40-42]. From this point of view, our findings may support possible targeted therapies with AgNano application.

It has been shown that *VEGFA* mRNA and protein are increased in the muscle during angiogenesis caused by repeated exercise [43-45]. Also, the mechanisms associated with bradycardia provide a signal for the enhancement of VEGFA expression, which is responsible for the myocardial angiogenesis [46]. We have demonstrated that AgNano at a concentration of 20 ppm administered to broiler chickens in the drinking water caused up-regulation of *VEGFA* expression in the heart on the mRNA and protein level. VEGFA modulates angiogenesis in dystrophic muscle [17,18] and in the heart [21,46]. To reveal whether AgNano might influence the heart angiogenesis in our experiment, resulting in the neovascularisation of the cardiac muscle, histological examination of the tissue should be performed.

Recent data indicate that regulation of *VEGFA* and *FGF2* expression occurs on the transcriptional as well as translational levels, depending on the tissue and cell type [8,33-35,47,48]. Our results support these findings, showing that *VEGFA* and *FGF2* expression can be regulated pre- and post-transcriptionally. Transcriptional and translational regulation of gene expression comprises the cascade of interactions between different biological molecules, including DNA, proteins, mRNAs and miRNAs [49-51]. Our results demonstrate that, in the case of *FGF2* expression in the muscle, regulatory mechanisms prevent the increase of the level of the protein product of the gene.

Experimental data show that *in ovo* treatment can provide a basis for muscle growth and stimulate coronary development [20,21]. However, some of our unpublished data suggest that AgNano injected *in ovo* at early stages of the embryonic development seem not to evoke long-lasting influence on gene expression, which is prolonged after the hatching period. Therefore, our aim was rather to observe whether *in ovo* injection could change the effect of AgNano distributed with drinking water. Answering this question, we assumed that indeed, *in ovo* injection up-regulated both the heart expression of *FGF2* as well as the muscle expression of *VEGFA* in broilers treated with drinking solution of 20 ppm of AgNano. However, the AgNano drinking solution itself (both concentrations) evoked up-regulation of *FGF2* expression in the muscle, without enhancing the effect of *in ovo* injection.

Changes in the expression of investigated genes on the protein level may lead to harmful or beneficial histological changes. When examining silver residues in organs after AgNano administration, our unpublished results have shown minor Ag deposition in the breast muscle tissue and very slight deposition in the hearts of birds treated with a 20-ppm solution of colloidal silver, compared to the control group. However, these results could not inform us regarding the form of Ag deposited in the tissues. Supporting data from histological and TEM examinations of these tissues are needed to elucidate whether AgNano or rather silver ions exert a direct action.

## Conclusions

AgNano application in poultry feeding can significantly influence the expression of *FGF2* and *VEGFA* genes on the mRNA and protein levels in growing chicken. *In ovo* injection of AgNano at early embryonic stages of the development can enhance the effect of AgNano, distributed as a drinking solution, on *FGF2* and *VEGFA* expression. On the protein level, AgNano influence FGF2 and VEGFA expression only in the heart, whereas in the muscle, the level of FGF2 and VEGFA protein remains unchanged. Supporting data from histological and TEM examinations of these tissues are needed to elucidate whether AgNano or rather silver ions exert a direct action on gene expression, and whether AgNano could evoke neovascularisation in the heart.

## Abbreviations

*ACTB*: Actin beta; AgNano: Nanoparticles of colloidal silver; ANOVA: Analysis of variance; *EEF1A2*: Elongation factor 1 alpha 2; ELISA: Enzyme-linked immunosorbent assay; FGF2: Basic fibroblast growth factor 2; SEM: Standard error of the mean; TEM: Transmission electron microscope; TP: Total protein; VEGFA: Vascular endothelial growth factor A.

## Competing interests

The authors declare that they have no competing interests.

## Authors’ contributions

AH carried out the molecular genetic studies and drafted the manuscript. ES conceived the study. LP participated in the design and supervised the experiment. FS participated in the statistical analysis. MG participated in the molecular studies and helped draft the manuscript. AC participated in the design and coordination and helped draft the manuscript. All authors read and approved the final manuscript.

## Authors’ informations

AH is a PhD and postdoc at the University of Copenhagen (UC), ES is a PhD, DSc, professor and head of department at Warsaw University of Life Sciences (WULS), LP is a PhD and postdoc at UC, FS is a PhD student at UC, MG is a PhD and postdoc at WULS, and AC is a DSc, professor and head of division at UC.
